# The safety and efficacy of hybrid ablation for the treatment of atrial fibrillation: A meta-analysis

**DOI:** 10.1371/journal.pone.0190170

**Published:** 2018-01-03

**Authors:** Yun-Qiu Jiang, Ying Tian, Li-Jun Zeng, Shu-Nan He, Zhi-Tao Zheng, Liang Shi, Yan-Jiang Wang, Yu-Xing Wang, Xian-Dong Yin, Xiao-Qing Liu, Xin-Chun Yang, Xing-Peng Liu

**Affiliations:** 1 Cardiac Arrhythmias Section, Heart Center, Beijing Chao-Yang Hospital, Capital Medical University, Beijing, China; 2 Department of Cardiology, Beijing Lu-He Hospital, Capital Medical University, Beijing, China; University of Tampere, FINLAND

## Abstract

**Introduction:**

Hybrid ablation, an emerging therapy that combines surgical intervention and catheter ablation, has become a viable option for the treatment of persistent atrial fibrillation. In this analysis, we aimed to evaluate the safety and efficacy of hybrid ablation, as well as compare the outcomes of one-step and staged approaches.

**Methods:**

We conducted a search in major online databases and selected the studies that met the inclusion criteria. The primary endpoint was defined as no episode of atrial fibrillation or atrial tachycardia lasting longer than 30 seconds without administration of antiarrhythmic drugs.

**Results:**

Sixteen studies including 785 patients (paroxysmal atrial fibrillation, n = 83; persistent atrial fibrillation, n = 214; long-standing persistent atrial fibrillation, n = 488) were selected. Average history of atrial fibrillation was (5.0±1.6) years. The pooled proportion of patients who were arrhythmia-free at the primary endpoint was 73% (95% CI, 64%–81%, Cochran’s Q, P<0.001; I^2^ = 81%). The pooled rate of severe short-term complications was 4% (95% CI, 2%–7%, Cochran’s Q, P = 0.01; I^2^ = 51%). The success rate after one-step procedures (69%) was lower than that after staged procedures (78%). The staged approach could ultimately prove to be safer, although complication rates were relatively low for both approaches (2% and 5%, respectively).

**Conclusions:**

Hybrid ablation is an effective and generally safe procedure. The current data suggest that staged hybrid ablation could be the optimal approach, as it is associated with a higher success rate and a seemingly lower complication rate. Additional randomized controlled trials are necessary to confirm these results.

## Introduction

Atrial fibrillation (AF) is the most common type of arrhythmia reported in clinical practice. As this condition is associated with an increased risk of major complications, such as thrombosis and stroke, finding optimal therapies for AF has long been a topic of interest among clinicians. Although the Cox Maze III procedure has been considered the gold standard for the surgical treatment of AF since 1991, [1] it has not been used extensively due to its complexity and invasive character. Pulmonary vein isolation (PVI), the standard catheter ablation procedure for paroxysmal AF (PrAF), has success rates exceeding 70% and acceptable complication rates [2]. However, neither epicardial ablation nor endocardial ablation alone can achieve a satisfactory result in patients with long-standing persistent AF (LSPsAF), making these patients prone to multiple repeat procedures and complications related to excessive atrial ablation. In recent years, surgeons have combined minimally invasive thoracoscopic surgery and percutaneous catheter ablation, creating a new procedure known as hybrid ablation, in an attempt to improve success rates and curb complication rates. A few systematic reviews have summarized the existing literature, focusing on aspects such as strategies for patient selection, periprocedural anticoagulation regimens, and ablation techniques [3,4]. However, the efficacy and safety of the hybrid ablation procedure have not been sufficiently studied. Factors that can influence the success rates and the incidence of perioperative complications are yet to be determined. To this end, we performed a pooled analysis and meta-analysis of data from existing studies and trials.

## Methods

### Search strategy and study selection

Our systematic literature search was performed according to MOOSE [5], Preferred Reporting Items for Systematic Reviews and Meta-Analyses (PRISMA)(S1 File), and Cochrane guidelines. We ran searches using EMBASE (1947–Oct. 2017) (S2 File), MEDLINE (1950–Oct. 2017) (S3 File), and the Cochrane library. Two authors (first and second author) independently reviewed all articles. The primary endpoint was defined as no episode of AF or atrial tachycardia longer than 30 seconds without administration of antiarrhythmic drugs. We selected only prospective studies in which all subjects in the treatment group underwent both epicardial and endocardial ablation, either simultaneously or separately. Additionally, the follow-up methods and results, including the number of patients who were arrhythmia-free at the end of the follow-up period, had to be clearly described. Studies were excluded if they did not distinctly report the follow-up outcomes or if they reported that epicardial ablation was performed by sternotomy. When several studies covered overlapping data, we chose the study including the greatest number of patients. The corresponding author made the final decision when the fist and the second author disagreed regarding the inclusion of an article or extracted data. The data were registered into a Microsoft Excel Office 2011 spread sheet.

### Risk of bias

Quality and bias of single-arm trials were assessed using a modified version of the Newcastle–Ottawa Scale (NOS) [6], a nine-point scale originally designed to assess risk of bias in cohort studies. Questions concerning control groups were excluded from the scale. Cohort studies were assessed using the original NOS scale. The first three questions assessed selection of the study population and representativeness of the study groups (age of patients, types and duration of AF). The following two questions evaluated how the studies determined exposure and assessed evidence that no similar procedures were previously performed. The remaining three points were awarded for outcome quality (Holter monitoring lasting at least 7 days as proof of sinus rhythm; SR), duration of follow-up (at least 12 months), and bias due to dropout or incomplete follow-up. A score of 5 or lower indicated a low-quality cohort study, while a score of 3 or lower was a marker of poor quality for single-arm trials.

### Data analysis

Data from all included studies were extracted into Stata (Version 14.0. College Station, TX, USA) and pooled using a random-effects model (S4 File). A 95% confidence interval (CI) was calculated. Statistical heterogeneity was assessed using Cochran’s Q test (χ2) and I^2^ method. In the Q test, a P value of <0.1 was deemed statistically significant. The I^2^ method was used to assess the degree of heterogeneity, with a score discrimination of 0%–40%, 30%–60%, 50%–90%, and 75%–100% indicating low, moderate, substantial, and considerable heterogeneity, respectively [7]. Funnel plots were constructed to explore publication bias. Incomplete or uneven plots indicated a high risk of publication bias.

## Results

### Study characteristics

**Fig 1:**
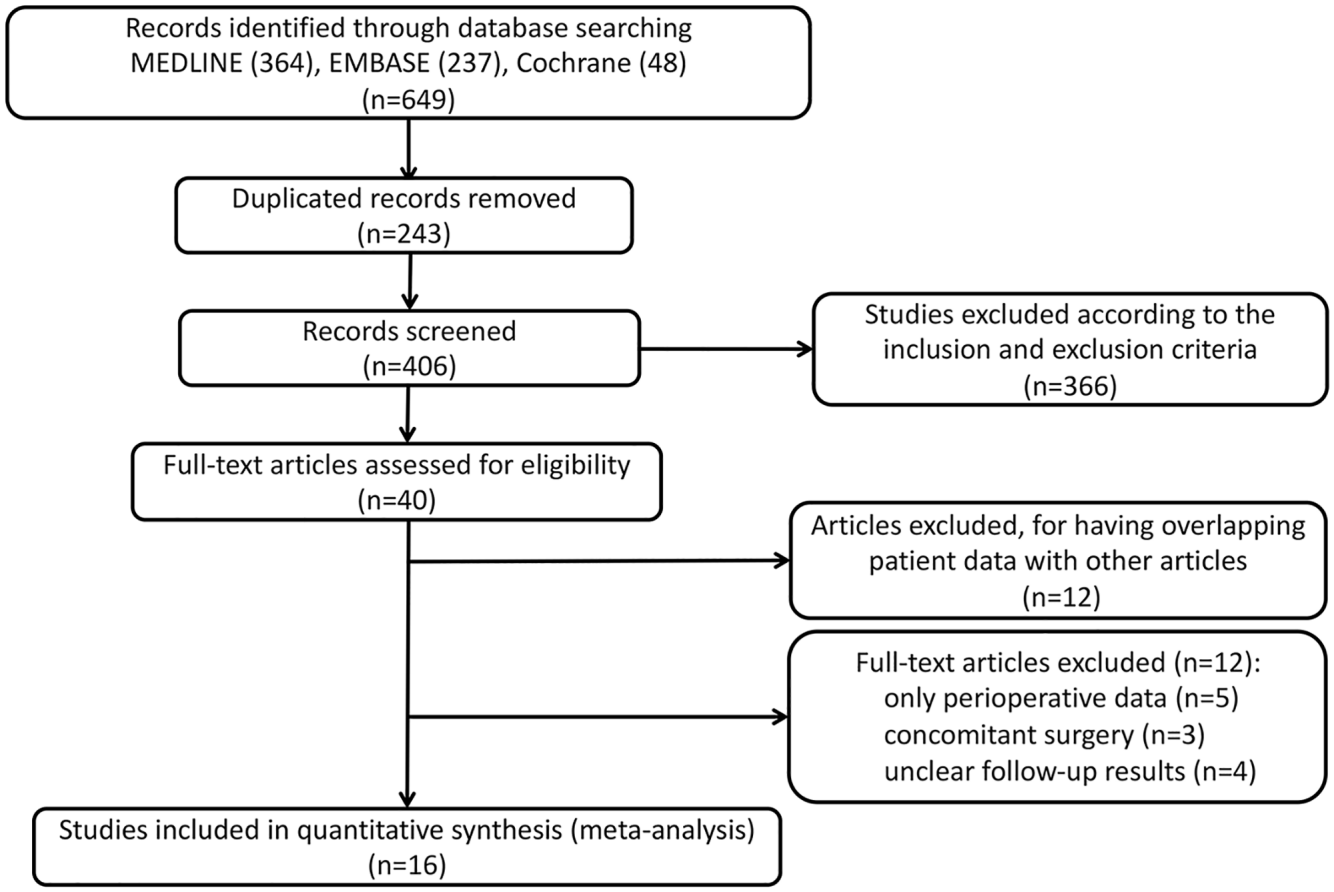
Flow diagram of identified studies.

A total of 649 studies were identified (Fig 1): 364 from MEDLINE, 237 from EMBASE, and 48 from Cochrane Library. Subsequently, 243 duplicate records were excluded. After reviewing titles and abstracts, we excluded 366 other studies according to the inclusion and exclusion criteria. Case reports, letters, animal experiments, and reviews were removed. Of the remaining records, 12 other studies with irrelevant or ambiguous endpoints were excluded, as were 12 studies with data previously reported in other articles. Sixteen studies were included after the final review, comprising three cohort studies and 13 single-arm trials.

A total of 785 patients from 16 studies were included (PrAF, n = 83; PsAF, n = 214; and LSPsAF, n = 488). Average history of AF was (5.0±1.6) years. The mean follow-up period was (21±11) months. Background information and baseline characteristics from all studies are summarized in Table 1. The three cohort studies did not have significant differences in any of the main baseline characteristics between groups (P>0.05). Methods and follow-up regimens used in the included studies are summarized in Table 2. At the end of the follow-up periods, 119 patients were “lost to follow-up”. Of the remaining 666 cases, 551 (82.7%) were in SR.

**Table 1:** Baseline characteristics and patient demographics.

Nr	Study	Pts	Type(s) of AF	Duration (Yrs)	LSPsAF	LSPsAF%	PsAF	PsAF%	PrAF	PrAF%	Failed RFCA(%)
1	Kiser(2011)	65	Pr, Ps, LSP	8	48	74	12	18	5	8	0
2	Mahapatra(2011)	15	Ps, LSP	5.4	6	40	9	60	0	-	100
3	Muneretto(2012)	36	Ps, LSP	4.2	28	78	8	22	0	-	0
4	La Meir(2012)	19	Pr, Ps, LSP	5	10	53	4	21	5	26	58
5	Bisleri(2013)	45	LSP	4.3	45	100	0	-	0	-	NR
6	La Meir(2013)	35	Pr, Ps, LSP	5	11	31	8	23	16	46	60
7	Pison(2013)	78	Pr, Ps, LSP	4	15	19	34	44	29	37	30
8	Gehi(2014)	101	Pr, Ps, LSP	5.9	37	37	47	47	17	17	36
9	Kurfirst(2014)	30	Ps, LSP	8.2	26	87	4	13	0	-	0
10	Kumar(2014)	7	Pr	2.8	0	-	0	-	7	100	100
11	Bulava(2015)	50	LSP	3.5	50	100	0	-	0	-	0
12	de Asmundis(2016)	64	Ps, LSP	5.2	43	67	21	33	0	-	30
13	Edgerton(2016)	24	LSP	6.8	24	100	0	-	0	-	NR
14	Gerask(2016)	76	Pr, Ps, LSP	5.2	60	79	12	16	4	5	NR
15	Zembala(2017)	90	Ps, LSP	4.5	51	57	39	43	0	-	43
16	Osmančík(2017)	50	Ps, LSP	2.7	34	68	16	32	0	-	0

Nr = number; Pts = patients; AF = atrial fibrillation; LSPsAF = long-standing persistent atrial fibrillation; PsAF = persistent atrial fibrillation; PrAF = paroxysmal atrial fibrillation; RFCA = radiofrequency catheter ablation.

**Table 2:** Methods and follow-up regimens of included studies.

Nr	Study	Follow-up	Surgical Approach	Staged/One-step	Energy Source	Repeat CA	LAA Removal (%)	NOS
1	Kiser(2011)	12	LAP	One-step	Unipolar	0	0	4
2	Mahapatra(2011)	21	Bilateral VATS	Staged	Bipolar	0	93	7
3	Muneretto(2012)	30	R-VATS	Staged	Unipolar	0	0	5
4	La Meir(2012)	12	R-VATS	One-step	Unipolar	0	0	6
5	Bisleri(2013)	28	R-VATS	Staged	Unipolar	0	0	5
6	La Meir(2013)	12	Bilateral VATS	One-step	Bipolar	0	43	7
7	Pison(2013)	24	R-VATS	One-step	Bipolar	10	45	5
8	Gehi(2014)	12	LAP	One-step	Unipolar	6	0	4
9	Kurfirst(2014)	7	Bilateral VATS	Staged	Bipolar	2	63	4
10	Kumar(2014)	40	R-VATS	One-step	Bipolar	1	0	3
11	Bulava(2015)	12	Bilateral VATS	Staged	Bipolar	1	84	5
12	de Asmundis(2016)	23	Bilateral VATS	One-step	Bipolar	14	73	5
13	Edgerton(2016)	24	LAP	One-step	Unipolar	0	0	3
14	Gerask(2016)	48	LAP	Both	Unipolar	12	0	5
15	Zembala(2017)	12	LAP	Both	Unipolar	1	0	5
16	Osmančík(2017)	20	R-VATS	Staged	Both	5	48	5

Nr = number; Repeat CA = repeat catheter ablation; LAA = left atrial appendage; VATS = video-assisted thoracoscopy; R = right side; L = left-side; LAP = laparoscope; NOS = Newcastle–Ottawa Scale.

### Overall meta-analysis

#### Efficacy

**Fig 2:**
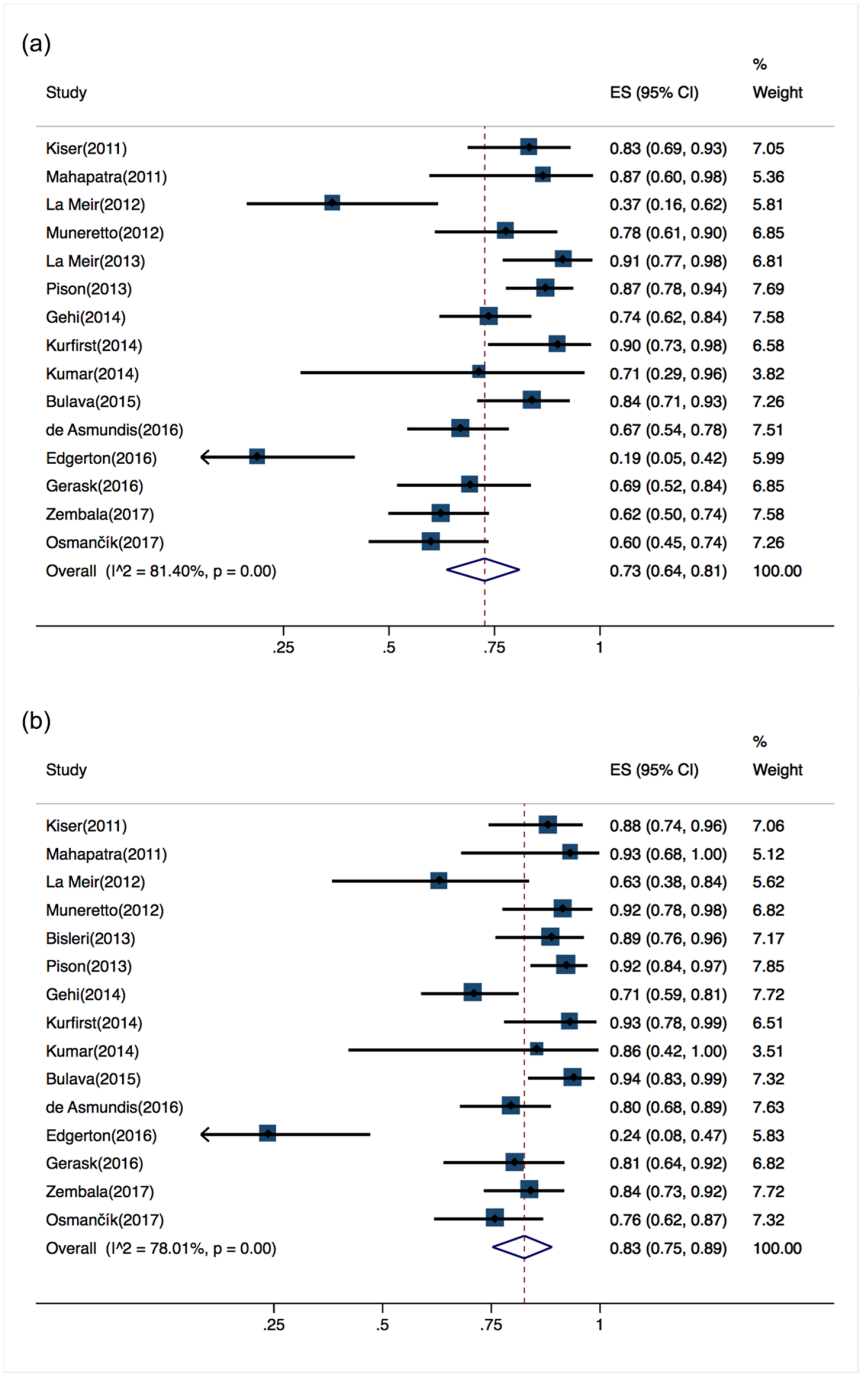
(a) Forest plot of the overall pooled proportion of arrhythmia-free patients without antiarrhythmic drugs (AADs) in all studies. (b) Forest plot of the overall pooled proportion of arrhythmia-free patients with AADs or repeat ablation procedures in all studies.

The most commonly used follow-up methods in the 16 included studies were 7-day Holter monitoring and implantable cardiac monitors. However, two studies [8,9] used mostly 24-hour Holter monitoring, while two others [10, 11] employed 24-hour Holter monitoring or 7-day continuous event monitoring with auto-trigger recorders at different follow-up visits. The pooled proportion of patients who underwent hybrid ablation and were arrhythmia-free at the end of follow-up without antiarrhythmic drugs (AADs) (the primary endpoint reported in 15 studies) was 73% (95% CI, 64%–81%), with a substantial risk of heterogeneity (Cochran’s Q, P<0.001; I^2^ = 81%) (Fig 2a). Only 10 of the 15 studies distinctly reported the proportion of arrhythmia-free patients with administration of AADs but no repeat ablations. The pooled proportion of these patients was 81% (95% CI, 69%–91%), with a considerable risk of heterogeneity (Cochran’s Q, P<0.001; I^2^ = 88%). The rest of the studies failed to specify the number of repeat ablation procedures that their participants underwent. Among the studies (n = 15) reporting the success rate with AADs, repeat catheter ablation (52/666), or both, the pooled success rate was 83% (95% CI, 75%–89%), with a substantial risk of heterogeneity (Cochran’s Q, P<0.001; I^2^ = 78%) (Fig 2b).

The clinical heterogeneity across the studies prompted us to perform a subgroup analysis of patients with LSPsAF. These data were available in seven studies (n = 168). The pooled success rate was 70% (95% CI, 51%–86%) at the primary endpoint (no arrhythmia without AADs), with a considerable risk of heterogeneity (Cochran’s Q, P<0.001; I^2^ = 90%).

#### Safety

**Fig 3:**
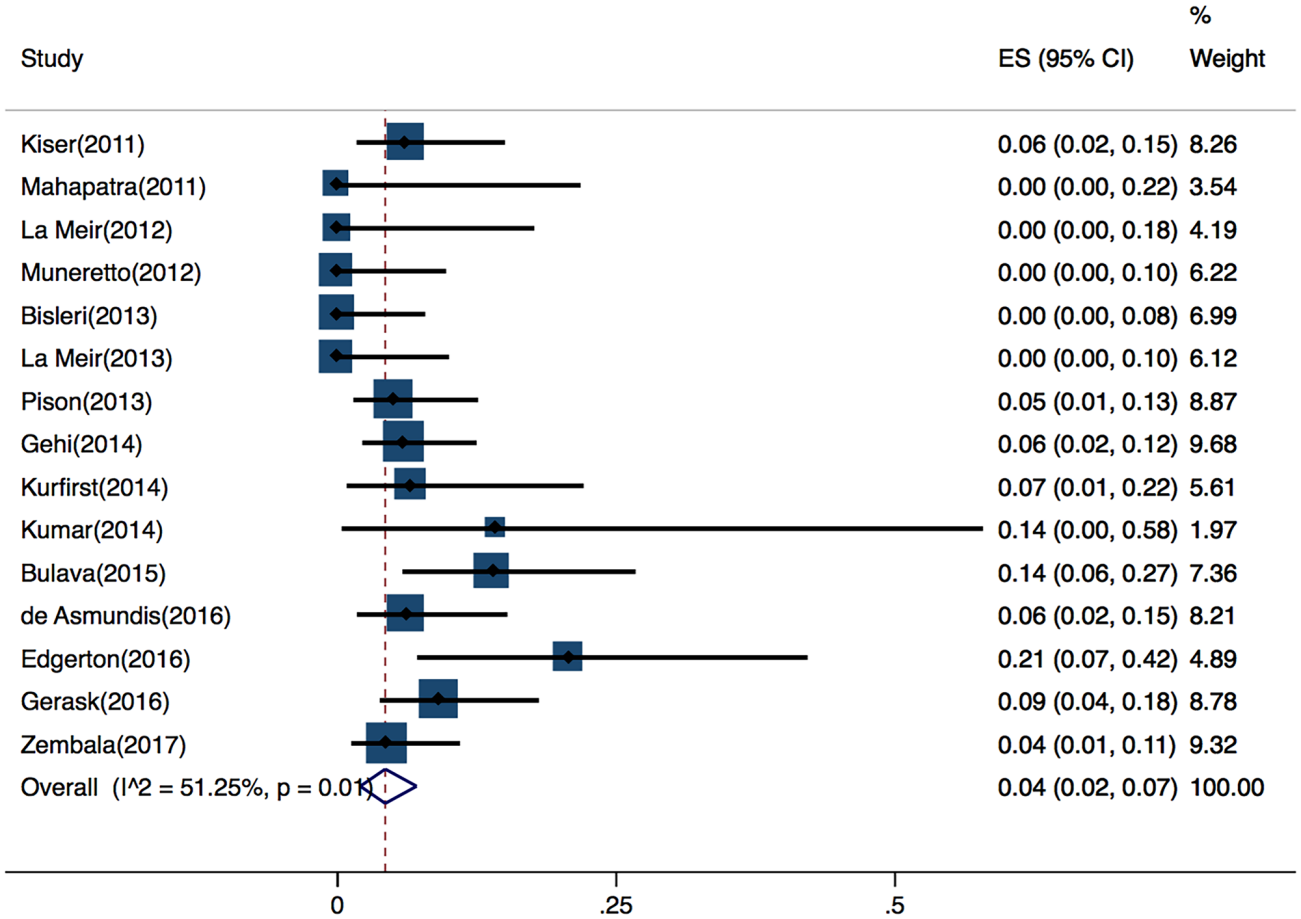
Forest plot of overall pooled rate of severe short-term complications.

The rates and types of severe short-term complications were reported in detail in15 of the 16 studies. Severe short-term complications were defined as death during the perioperative period, conversion to sternotomy, atrial-esophageal fistula, permanent phrenic nerve injury, pericardial tamponade, pericardial effusion requiring drainage, myocardial infarction, stroke, hemothorax, and other major bleeding events. The pooled proportion of patients who developed severe short-term complications was 4% (95%, CI 2%–7%), with a moderate risk of heterogeneity (Cochran’s Q, P = 0.01; I^2^ = 51%) (Fig 3).

### Subgroup analysis

#### Energy sources

**Fig 4:**
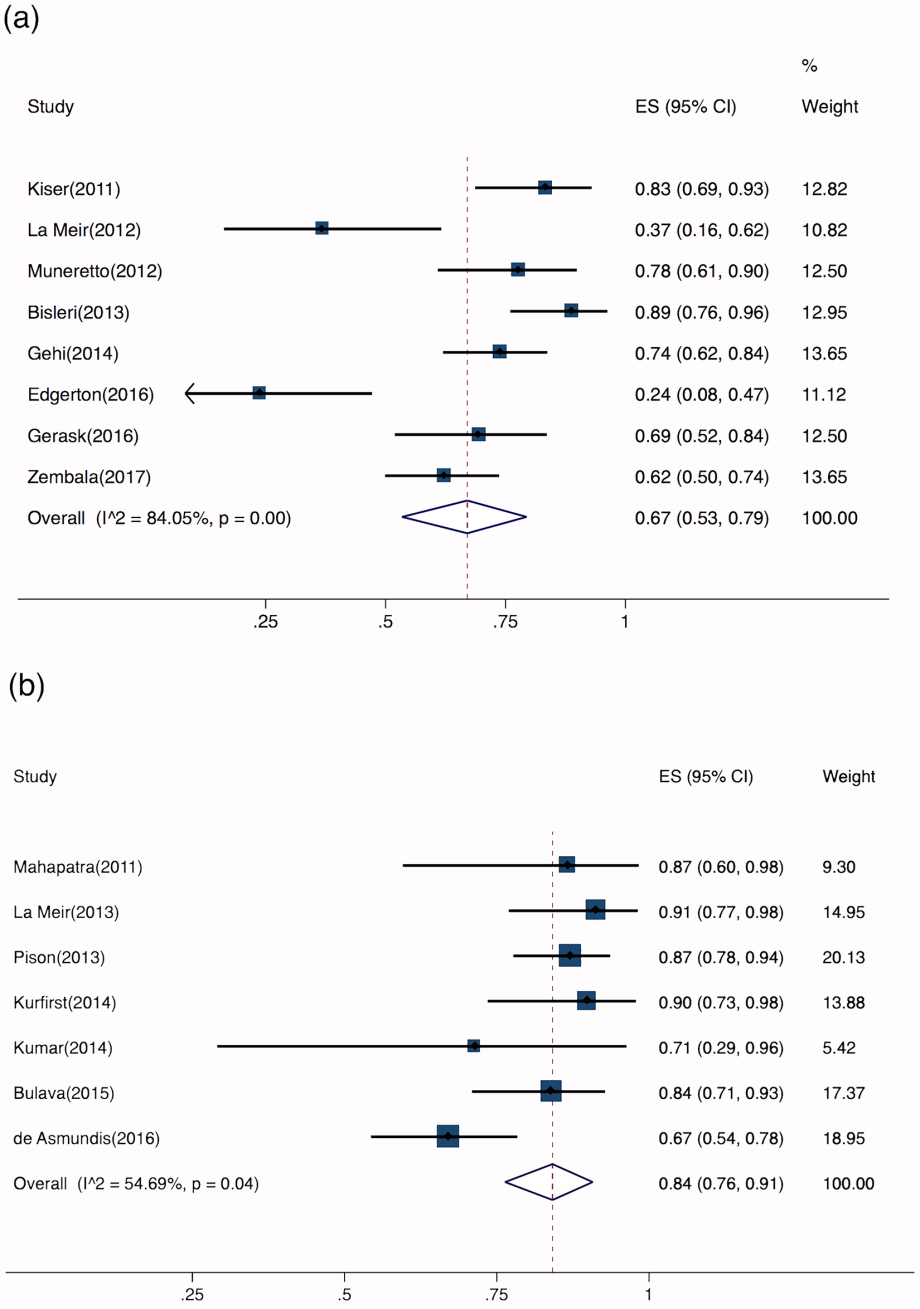
Individual forest plots of subgroups. a. Forest plot of studies that used unipolar radiofrequency (RF) energy; b. Forest plot of studies that used bipolar RF energy.

All 16 studies reported the energy sources used for surgical ablation. Epicardial ablation was performed using unipolar radiofrequency (RF) energy in eight studies (n = 411). Bipolar RF was used in seven studies (n = 324). One study adopted a unipolar/bipolar ablation device and was excluded from this subgroup analysis [12]. The pooled success rate after epicardial unipolar RF ablation was 67% (95% CI, 53%–79%), with a substantial risk of heterogeneity (Cochran’s Q, P<0.001; I^2^ = 84%) (Fig 4a). After bipolar RF ablation, the success rate was 84% (95% CI, 76%–91%), with a moderate risk of heterogeneity (Cochran’s Q, P = 0.04; I^2^ = 55%) (Fig 4b).

#### Staged versus one-step ablation procedure

**Fig 5:**
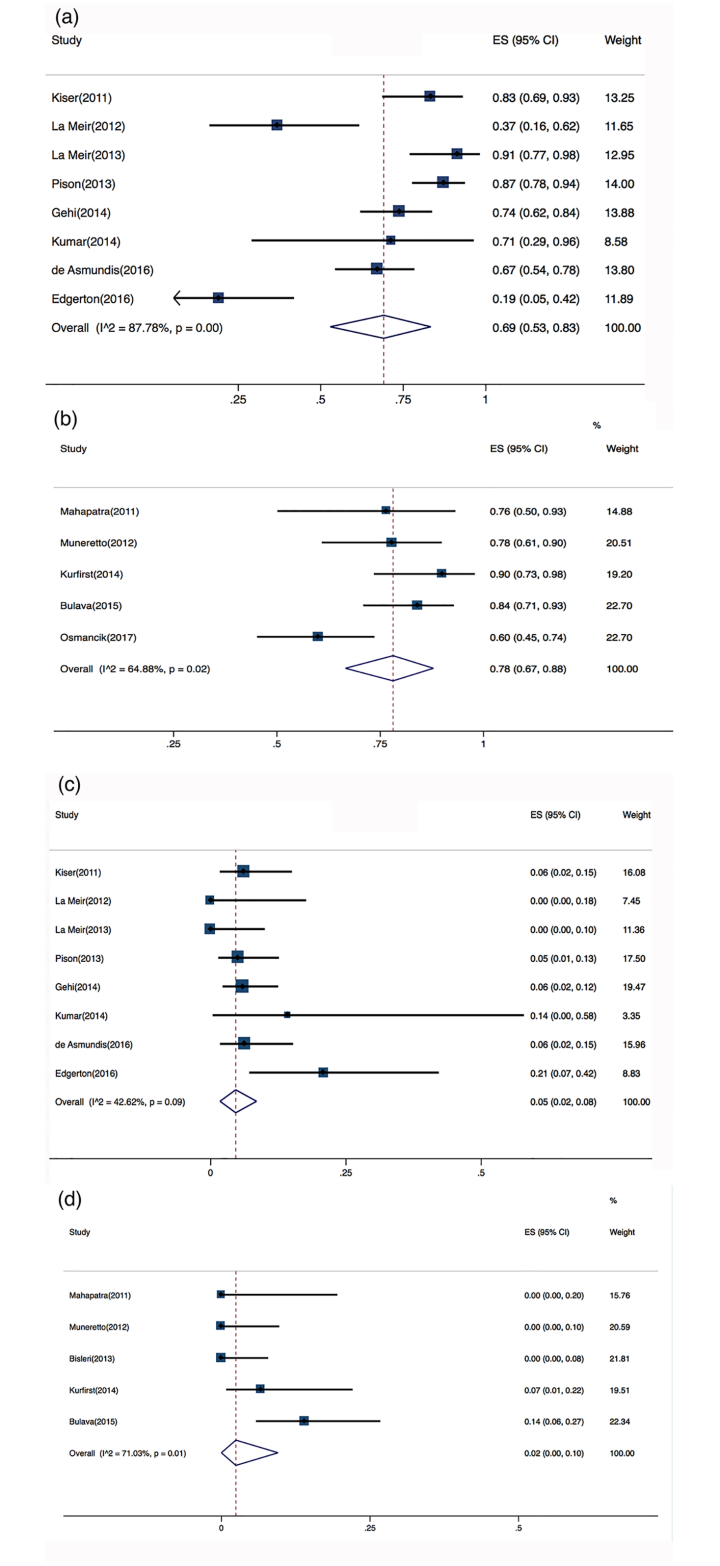
Individual forest plots of subgroups. a. Forest plot of one-step hybrid ablation: percentage of arrhythmia-free patients; b. Forest plot of staged hybrid ablation: percentage of arrhythmia-free patients; c. Forest plot of one-step hybrid ablation: rate of short-term complications; d. Forest plot of staged hybrid ablation: rate of short-term complications.

Six of the 16 studies adopted a staged surgical approach, but only five of these reported the proportion of patients in SR at the primary endpoint (n = 181). The time interval between the two procedures ranged from 3–4 days [10] to 3 months [13]. Eight trials reported using a one-step procedure (n = 411). Gersak et al [14] and Zembala et al [15] used both one-step and staged approaches. In a related article, Zembala et al [8] attributed the choice of intervention to reimbursement issues. At the primary endpoint, the pooled success rate after a one-step procedure was 69% (95% CI, 53%–83%), with a considerable risk of heterogeneity (Cochran’s Q, P<0.001; I^2^ = 88%) (Fig 5a). The success rate after a staged procedure was 78% (95% CI, 67%–88%), with a substantial risk of heterogeneity (Cochran’s Q, P = 0.02; I^2^ = 65%) (Fig 5b).

The pooled complication rate after a one-step procedure was 5% (95% CI, 2%–8%), with a moderate risk of heterogeneity (Cochran’s Q, P = 0.09; I^2^ = 43%) (Fig 5c). The complication rate after a staged procedure was 2% (95% CI, 0%–10%), with a substantial risk of heterogeneity (Cochran’s Q, P = 0.01; I^2^ = 71%) (Fig 5d). The two studies (n = 193) that used both staged and one-step procedures but did not report complications separately were excluded from this subgroup analysis.

#### Previous catheter ablation (CA)

**Fig 6:**
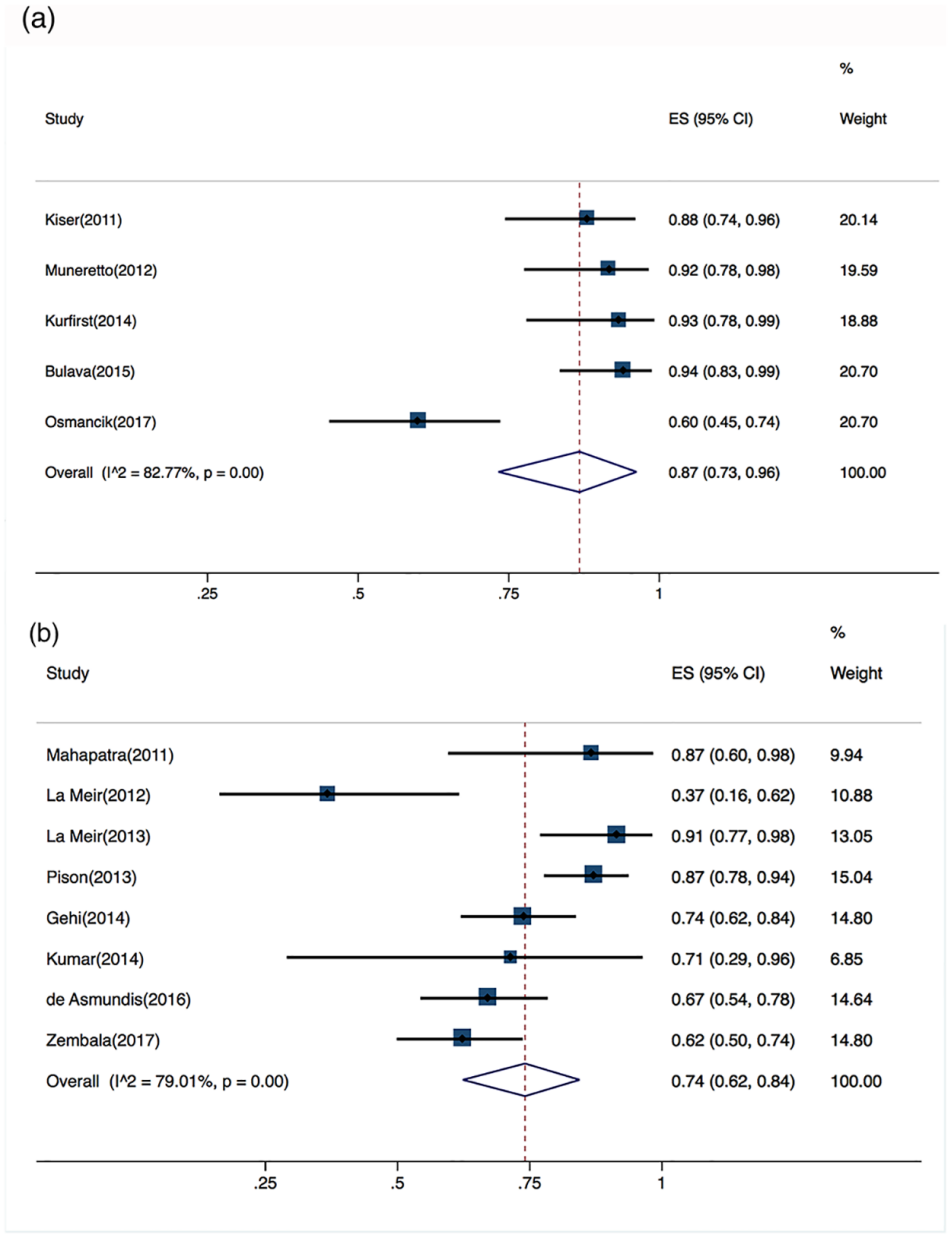
Individual forest plots of subgroups. a. Forest plot of patients with no previous percutaneous catheter ablation (PCA): percentage of arrhythmia-free patients; b. Forest plot of patients with previous PCA: percentage of arrhythmia-free patients.

Three studies that did not report whether the patients underwent previous catheter ablation procedures were excluded from the subgroup analysis. Across the five studies (n = 231) that included only patients who had not experienced previous CA, the pooled success rate without AADs was 87% (95% CI, 73%–96%), with a substantial risk of heterogeneity (Cochran’s Q, P<0.001; I^2^ = 83%) (Fig 6a). Across the eight studies (n = 409) that included patients who had experienced previous CA (for AF, atrial flutter, or supraventricular tachycardia, reported in 30%-100% of the patients), the pooled success rate without AADs was 74% (95% CI, 62%–84%), with a substantial risk of heterogeneity (Cochran’s Q, P<0.001; I^2^ = 79%) (Fig 6b).

#### Removal of left atrial appendage (LAA) and complications

Removing the LAA could be associated with a higher incidence of perioperative complications, particularly hemorrhaging complications. None of the included studies utilized LAA occlusion devices. Across the seven studies (n = 322) that reported excision, ligation, or clip occlusion of the LAA during epicardial ablation (reported in 43%-100% of the patients), the pooled complication rate was 5% (95% CI, 1%–9%), with a moderate risk of heterogeneity (Cochran’s Q, P = 0.10; I^2^ = 46%). Across the nine studies (n = 463) in which the LAA remained untouched, the pooled complication rate was 4% (95% CI, 1%–8%), with a moderate risk of heterogeneity (Cochran’s Q, P = 0.01; I^2^ = 59%).

### Sensitivity analysis

Two studies were excluded from the sensitivity analysis for compelling reasons. Kumar et al [16] used an essentially different methodology compared to the other studies. Only some of the pulmonary veins were isolated by epicardial ablation in this study, while cryoablation was used in the rest of the pulmonary veins. The trial conducted by Edgerton et al [17] scored three points on the NOS and was excluded from the sensitivity analysis due to low study quality.

After excluding those two studies (n = 31), the pooled success rate without AADs across the studies was 76% (95% CI, 68%–83%, Cochran’s Q, P<0.001; I^2^ = 75%), while the pooled success rate with AADs or repeat CAs was 85% (95% CI, 80%–90%, Cochran’s Q, P<0.001; I^2^ = 59%). Funnel plots showed a moderate risk of publication bias, as the studies were equally distributed on both sides of the central axis (S1 Fig).

The recalculated incidence of severe complications was 4% (95% CI, 2%–7%, Cochran’s Q, P = 0.04; I^2^ = 46%).

## Discussion

### Main findings

Hybrid ablation has emerged as a potentially effective treatment option for patients who suffer from LSPsAF. Although several traditional reviews and a few systematic reviews have discussed hybrid ablation, to date, this is the first meta-analysis focusing on the safety and efficacy of this novel procedure. We conducted a review of the relevant literature, which consisted mostly of observational studies, as anticipated. We analyzed the success rates and complication rates in the 16 studies we ultimately selected for inclusion. Subgroup analysis and sensitivity analysis were also conducted.

#### Efficacy of hybrid ablation

In the 16 included studies, 82.7% (551/666) of patients were in sinus rhythm at the end of the follow-up period. The pooled proportion of patients in SR without administration of AADs was 73%. This was higher than the success rate (66%) previously reported in a systematic review that simply obtained an average of the SR rates in multiple studies. The pooled success rate with AADs or repeat catheter ablation(s) was 83%, which was also higher than the 78% rate reported in the aforementioned systematic review [4]. This difference could be attributed to the superiority of meta-analyses in summarizing scientific results. Certain differences in article selection could also explain the discrepancy between the success rates. Trials conducted in different periods of time by the same researcher were included in our study, but excluded from the review article by Vroomen et al [4]. Meanwhile, we excluded some articles with a high risk of overlap between study populations.

Few studies have directly compared the success rates of hybrid ablation to those of other therapies. Mahapatra et al [10] matched 15 consecutive patients who underwent hybrid ablation to 30 patients who underwent a repeat CA. After a follow-up of (20.7 ± 4.5) months, 13/15 (86.7%) patients were free from AF and off AADs after hybrid ablation, while the proportion of patients without arrhythmia and off AADs after repeat CA was only 16/30 (53.3%). This study demonstrated higher success rates after hybrid ablation compared with a repeat CA, suggesting that hybrid ablation could represent a last-resort therapy for patients with LSPsAF or other indicators of an unfavorable prognosis, such as previously failed CA.

Lesion transmurality is crucial for achieving complete and durable PVI. A variety of energy sources, including RF, microwave, laser, high frequency ultrasound, and cryothermy, have been used for the ablation of AF [18]. Experimental animal models [2,18] have shown that surgical ablation using bipolar RF clamps is the most reliable method of creating circumferential transmural lesions in the pulmonary veins. Clinical trials have confirmed this result. In a review article, the success rate ranged from 85.7% to 92% in studies employing bipolar RF energy and from 36.8% to 88.9% in those utilizing unipolar RF energy [19]. Our meta-analysis reached similar conclusions. The pooled proportion of patients in SR was 84% after bipolar ablation and 67% after unipolar RF ablation.

Patients who had undergone previous CA had a lower treatment success rate (74%) than those who had not (87%). This discrepancy could be explained by the fact that previously failed ablation is suggestive of challenging cases with a stronger tendency for PV reconnection after ablation and an atrial substrate that promotes AF maintenance. The mechanisms of AF recurrence might be different in these patients [20].

#### Safety of the procedure

Patient safety has always been a top priority for cardiac surgeons and electrophysiologists. The hybrid ablation procedure was designed to decrease the high rate of complications associated with the traditional cut-and-sew method, while ensuring a relatively high success rate. The overall rate of short-term severe complications in our meta-analysis was 4%. This was lower than the rate previously reported by Boersma et al [21] (9.8%) and Pokushalov et al [22] (25%) in epicardial only (mini-maze) ablation procedures, but comparable to the 3.6% rate reported by Wang et al [23]. As complications associated with hybrid ablation are reportedly mostly surgery-related, it is safe to conclude that the rate of severe complications associated with this procedure is no higher than the complication rate of stand-alone thoracoscopic surgical ablation. Although several authors reported no complications associated with the hybrid procedure in small to medium-sized population samples [10, 24–26], avoiding certain life-threatening complications remains a challenge for clinicians.

Atrial-esophageal fistula is a rare but life-threatening condition, which can be difficult to diagnose and can lead to death despite active treatment. Both Kiser et al [27] (two cases) and Gehi et al [9] (one case) reported the occurrence of atrial-esophageal fistulas and death after the use of the transdiaphragmatic surgical approach. Unipolar RF was the energy source for epicardial ablation in both of these studies. This complication could be attributed to the fact that bipolar RF clamps direct the ablation energy exclusively toward the pulmonary veins, while unipolar devices inevitably expose the esophagus to heat radiation during the ablation.

Currently, RF ablation can be performed by either a thoracoscopic or a transdiaphragmatic approach. Given the nature of epicardial ablation, the possibility of perioperative cardiac tamponade is low, since the pericardium is open during the surgical procedure [11]. Only one case of cardiac tamponade was reported (on day 24 after surgery) across the 11 studies that adopted the thoracoscopic approach in epicardial ablation. The five studies (n = 356) that adopted the transdiaphragmatic approach reported five cases of cardiac tamponade. The incidence of this complication across the five studies was 1.4% (5/356), suggesting that the transdiaphragmatic approach could carry a higher risk of cardiac tamponade. Further investigation is needed to identify the underlying causes.

Excision or ligation of the LAA during the procedure did not seem to influence the perioperative complication rate (4% with ligation vs. 5% with untouched LAA), although these steps can theoretically increase the odds of short-term hemorrhagic complications. Routine excision of the LAA during hybrid ablation procedures appears to be relatively safe.

As surgical centers continue to adopt hybrid ablation as a treatment option for persistent AF, safety remains the primary focus of clinicians. It is unreasonable to expose patients to a high risk of surgical complications, especially since hybrid ablation was designed to avoid the risks associated with open heart surgery. Further research is necessary to identify the best means of avoiding severe complications.

### One-step or staged procedure

By definition, the hybrid procedure combines thoracoscopic epicardial ablation with a catheter ablation procedure. However, whether a two-step procedure is superior to a one-step approach is yet to be determined. The subgroup analysis showed that the pooled success rate at the primary endpoint was lower after one-step procedures (69%) than after staged procedures (78%). The blanking period following PVI, during which the epicardial lesions mature and stabilize, may have contributed to this difference in outcomes. Reimbursement issues also played a role in the choice of procedure, as in some cases surgical decisions were shaped by reimbursement policies rather than medical considerations [8]. Safety outcomes favor the staged approach (2%), although the complication rates are relatively low with both approaches (2% vs. 5%). Administration of anticoagulants (heparin) during CA can theoretically lead to higher incidence of hemorrhagic complications. Prolonged procedural time is a risk factor for complications as well. Although it can be partly attributed to the learning curve of surgical teams, the 2%-5% complication rate is still far from satisfactory. Further refinement of surgical techniques is necessary. The current data suggest that staged hybrid ablation could be the optimal approach, due to its higher success rate and a seemingly lower complication rate.

### Endpoints for ablation

As hybrid ablation is still an emerging therapy, guidelines for its administration are yet to be established. There are no well-established standards for determining an endpoint for hybrid ablation. Few analyses have compared success rates between patients who achieved SR spontaneously during ablation and patients who had cardioversions after the procedure. De Asmundis et al [28] reported that conversion to SR during ablation did not improve clinical outcomes after an average follow-up time of 19 months (*P* = 0.47). To date, there is no evidence that achieving SR during ablation is associated with a better prognosis following hybrid ablation.

## Limitations

Due to the novelty of this procedure, the existing studies discussing hybrid ablation are mostly single-arm trials without control groups. Thus, there is no reliable comparison between the hybrid procedure and other therapies that are performed routinely. Furthermore, there were some differences in ablation sites and surgical techniques across studies discussing the hybrid procedure. We have also noticed a few differences in methodology between studies. We excluded those with extreme deviations from the sensitivity analysis to guard against bias.

## Conclusions

Hybrid ablation has proven to be an effective and generally safe procedure for the treatment of AF. The current data suggest that staged hybrid ablation could be the optimal approach, as it is associated with a higher success rate and a seemingly lower complication rate. The sensitivity analysis confirmed the stability of the analyzed data. However, additional randomized controlled trials are necessary to confirm our results.

## Supporting information

S1 Fig Funnel plots for the primary endpoints in the included studies. Studies are equally distributed on both sides of the central axis, indicating a moderate risk of publication bias.
(TIF)

S1 File PRISMA 2009 checklist. (DOC)

S2 File Search strategy for embase. (DOCX)

S3 File Search strategy for pubmed. (DOCX)

S4 File Raw data in stata software. (ZIP)
