# Supplementary figures and images for: The safety and efficacy of hybrid ablation for the treatment of atrial fibrillation: A meta-analysis
Source: PLoS One. 2018 Jan 3;13(1):e0190170. doi: 10.1371/journal.pone.0190170 (PMC5752005; doi:10.1371/journal.pone.0190170)

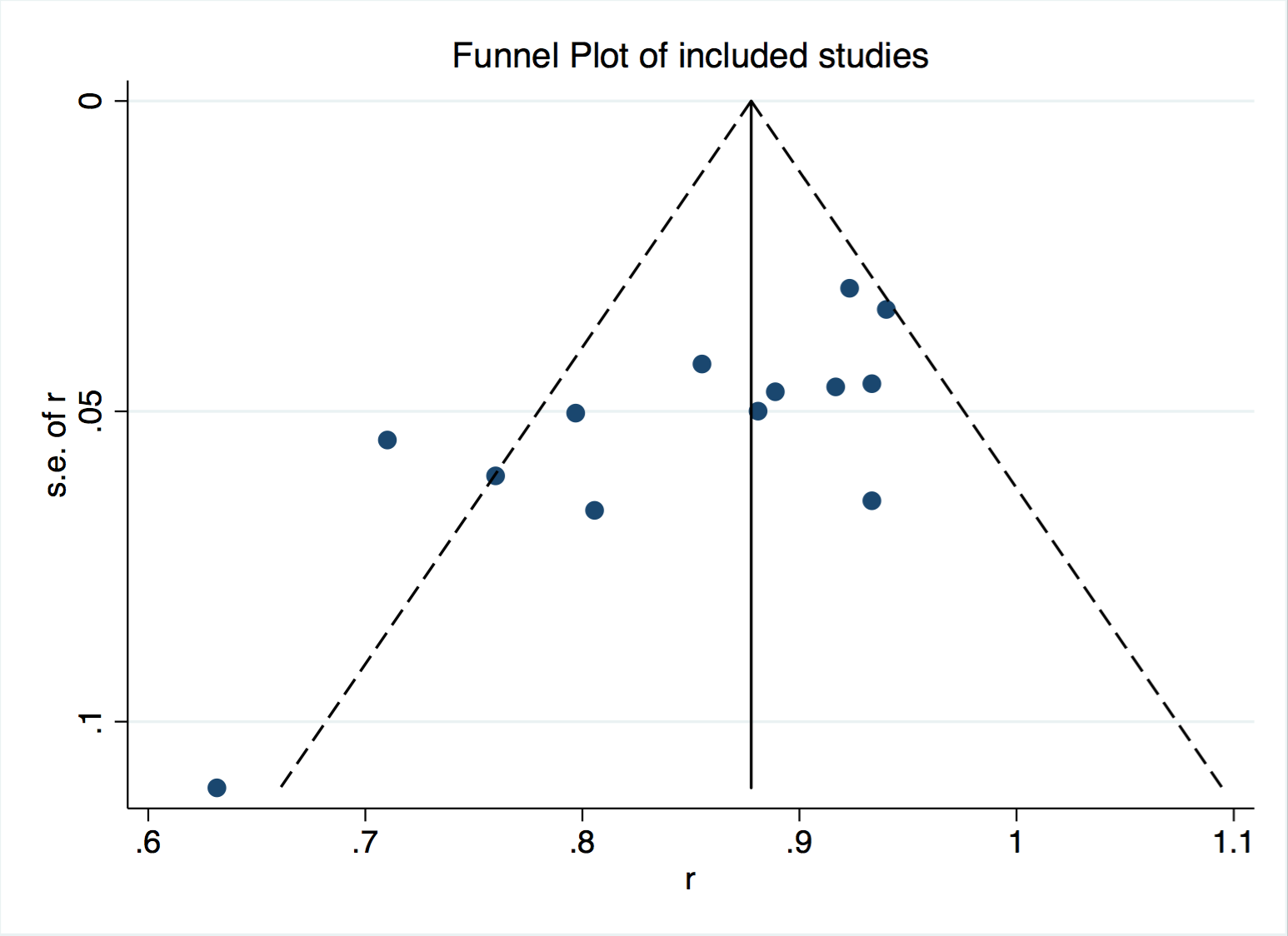

Supplement: S1 Fig — Studies are equally distributed on both sides of the central axis, indicating a moderate risk of publication bias. (TIF) [file pone.0190170.s001.tif]
