# Supplementary material for: The safety and efficacy of hybrid ablation for the treatment of atrial fibrillation: A meta-analysis
Source: PLoS One. 2018 Jan 3;13(1):e0190170. doi: 10.1371/journal.pone.0190170 (PMC5752005; doi:10.1371/journal.pone.0190170)
Supplement: S2 File — (DOCX) [file pone.0190170.s003.docx]

'atrial fibrillation'/exp AND ('catheter ablation'/exp AND ('endocardium' OR

'endocardium'/exp OR endocardium) OR ('pericardium'/exp OR pericardium AND ('minimally invasive surgery'/exp OR 'minimally invasive surgical procedure' OR 'minimally invasive surgical procedures')) OR 'hybrid procedure' OR 'hybrid surgery' OR 'hybrid ablation' OR 'hybrid therapy' OR 'hybrid treatment' OR 'hybrid approach' OR 'convergent procedure' OR 'convergent ablation' OR 'convergent surgery' OR 'convergent therapy' OR 'convergent treatment' OR 'simultaneously ablation' OR 'thoracoscopic ablation' OR 'comprehensive procedure' OR 'comprehensive ablation') AND ('single-arm' OR 'single arm' OR 'sinus rhythm'/exp OR 'randomized controlled trial'/exp OR 'cohort analysis'/exp)
