# Supplementary material for: The safety and efficacy of hybrid ablation for the treatment of atrial fibrillation: A meta-analysis
Source: PLoS One. 2018 Jan 3;13(1):e0190170. doi: 10.1371/journal.pone.0190170 (PMC5752005; doi:10.1371/journal.pone.0190170)
Supplement: S3 File — (DOCX) [file pone.0190170.s004.docx]

**Pubmed Search Strategy**

| **Search** | **Add to builder** | **Query** | **Time** |
| --- | --- | --- | --- |
| #18 | Add | Search **(#17 AND #3 AND (#7 OR #8 OR #10))** | 02:08:33 |
| #17 | Add | Search **(#11 OR #12 OR #15 OR #16)** | 02:02:40 |
| #16 | Add | Search **cohort study OR cohort studies** | 01:57:35 |
| #15 | Add | Search **randomized controlled** | 01:57:15 |
| #12 | Add | Search **sinus rhythm** | 01:54:07 |
| #11 | Add | Search **single arm trail*** | 01:53:26 |
| #10 | Add | Search **((Hybrid procedure) OR (hybrid surgery) OR (hybrid ablation) OR (hybrid therapy) OR (hybrid treatment) OR (hybrid approach) OR (convergent procedure) OR (convergent ablation) OR (convergent surgery) OR (convergent therapy) OR (convergent treatment) OR (simultaneously ablation) OR (thoracoscopic ablation) OR (comprehensive procedure) OR (comprehensive ablation))** | 01:51:40 |
| #8 | Add | Search **((("Pericardium"[Mesh]) OR Pericardium)) AND "Minimally Invasive Surgical Procedures"[Mesh]** | 01:05:23 |
| #7 | Add | Search **("Catheter Ablation"[Mesh]) AND (((Endocardium OR Endocardiums) OR "Endocardium"[Mesh]))** | 01:02:58 |
| #6 | Add | Search **((Endocardium OR Endocardiums) OR "Endocardium"[Mesh])** | 01:02:09 |
| #3 | Add | Search **"Atrial Fibrillation"[Mesh]** | 01:01:08 |
| #5 | Add | Search **"Catheter Ablation"[Mesh]** | 00:59:06 |
